# Automated Scanning Probe Tip State Classification without Machine Learning

**DOI:** 10.1021/acsnano.3c10597

**Published:** 2024-01-09

**Authors:** Dylan Stewart Barker, Philip James Blowey, Timothy Brown, Adam Sweetman

**Affiliations:** The School of Physics and Astronomy, Bragg Centre for Materials Research, The University of Leeds, Leeds LS2 9JT, United Kingdom

**Keywords:** scanning tunneling microscopy (STM), scanning probe microscopy (SPM), atomic resolution, machine learning, in situ tip conditioning, cross-correlation, automation

## Abstract

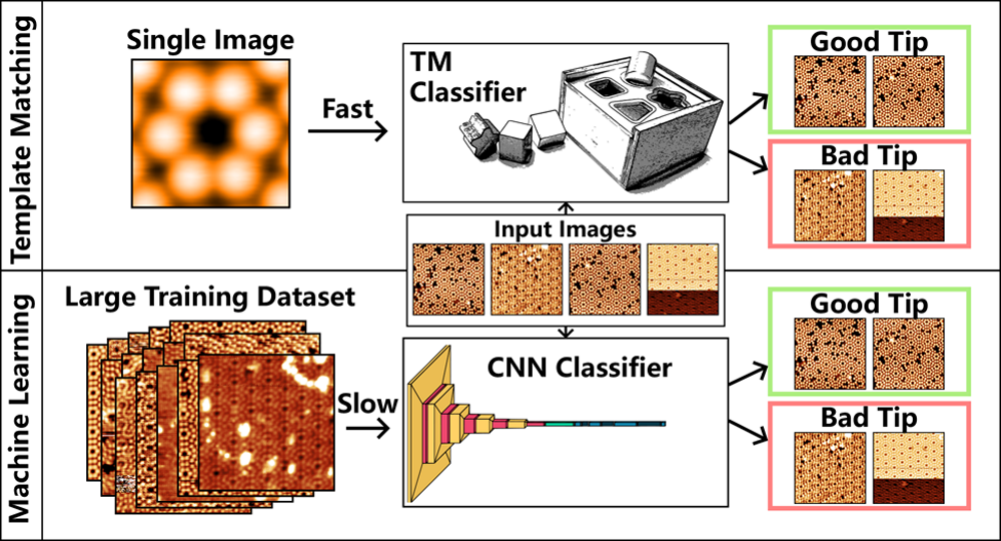

The manual identification and in situ correction of the state of the scanning probe tip is one of the most time-consuming and tedious processes in atomic-resolution scanning probe microscopy. This is due to the random nature of the probe tip on the atomic level, and the requirement for a human operator to compare the probe quality via manual inspection of the topographical images after any change in the probe. Previous attempts to automate the classification of the scanning probe state have focused on the use of machine learning techniques, but the training of these models relies on large, labeled data sets for each surface being studied. These data sets are extremely time-consuming to create and are not always available, especially when considering a new substrate or adsorbate system. In this paper, we show that the problem of tip classification from a topographical image can be solved by using only a single image of the surface along with a small amount of prior knowledge of the appearance of the system in question with a method utilizing template matching (TM). We find that by using these TM methods, comparable accuracy and precision can be achieved to values obtained with the use of machine learning. We demonstrate the efficacy of this technique by training a machine learning-based classifier and comparing the classifications with the TM classifier for two prototypical silicon-based surfaces. We also apply the TM classifier to a number of other systems where supervised machine learning-based training was not possible due to the nature of the training data sets. Finally, the applicability of the TM method to surfaces used in the literature, which have been classified using machine learning-based methods, is considered.

## Introduction

Atomic-resolution scanning probe microscopy (SPM) has revolutionized our ability to investigate nanoscale phenomena^1−3^ and manipulate matter with exceptional precision.^4−8^ Central to the success of SPM techniques is the quality and sharpness of the probe tip, which directly influences the resolution, sensitivity, and reliability of measurements. The manual in situ preparation of probe tips is a labor-intensive and time-consuming process, which poses a challenge to the efficiency and reproducibiltiy of SPM experiments, making it difficult to meet the growing demand for high-throughput SPM experiments. The ability to automate tip preparation is therefore desirable, as it would allow for operators to use their time elsewhere or assist in fully autonomous experimentation.

The main hurdle to overcome in producing a system for the automatic in situ preparation of tips is the classification of the state of the tip itself. This is usually carried out by an operator through comparisons between the expected surface structure and a few lines of a topograph while scanning, with the final decision being entirely based on the operator’s experience. It is also possible to use “inverse imaging” to characterize a tip, whereby the tip is scanned over a high aspect ratio surface feature, such as an adsorbed carbon monoxide (CO) molecule^9^ or a surface adatom,^10^ to image the shape of the probe apex. This difficulty in classification is specific to SPM methods and is not normally a consideration for other atomic-resolution methods, such as transmission electron microscopy (TEM). Recently, there has been great interest in the possibility of replacing human operators with trained machine learning (ML) models for tasks such as image evaluation, especially considering the successes in this area with handling complex problems in recent years.^11,12^

It has previously been shown that convolutional neural networks (CNNs) can be used to create models that are able to accurately classify tip states on multiple surfaces in both binary classifications (e.g., between “sharp” and “double” tip on H:Si(100))^13^ and multilabel classifications with multiple desirable tip states on H:Si(100), Au(111), and Cu(111).^14^ Advancements have also been made in ML as applied to SPM in increasing the speed of classifications by using partial scans on H:Si(100)^15^ or *I*(*V*) spectra on Au(111).^16^ In addition, full autonomous experiments have been conducted using ML-based classifiers, which are able to distinguish between various features present on a surface and react to each accordingly. This approach has been used for both lithography on H:Si(100)^17^ and data collection on Ag(100).^18^ CNNs have also been used to classify images for use in automated tip functionalization,^19^ specifically allowing for CO molecules to be picked up from a Cu(111) surface and for the resultant tip quality to be assessed via scanning tunneling microscopy (STM) imaging of other adsorbed CO molecules. Nevertheless, machine learning exhibits several drawbacks as an image classification technique. For example, it is difficult to implement and train and has issues related to data sets (such as insufficiently sized data sets, inherent biases, and inaccurate labeling); it is also difficult to comprehensively discern the knowledge acquired by the model. Factors such as these limit its general applicability to routine SPM operation, necessitating a substantial number of labeled data sets and requiring a high level of expertise for implementation. Some attempts have been made to address the problem of undesirable probe tips without the use of ML, with tip state classification attempts on highly oriented pyrolytic graphite (HOPG) in ambient conditions using an image analysis method known as the universal similarity metric.^20^ However, this method was found to perform poorly when analyzing STM images.^21^

In this paper, we present an alternative method for automating tip state classification using template matching (TM) methods, wherein input images are classified by comparison to a specific template, whether that be a reference image for cross-correlation (CC) or a perfect circle for circularity measurement. This is demonstrated using multiple prototypical surfaces imaged using STM. The TM-based classifier functions under various rotations and requires only a single image to complete a classification, contrary to the large data sets needed for ML. Its performance is compared to an ML-based classifier using CNNs (as was used in previous examples of tip classification) and to classifications performed by human operators. We highlight the limitations and advantages of both techniques and discuss them in the context of current state-of-the-art automated STM.

## Effect of Tips on Imaging

Topographical scans can appear with a large variety of image contrasts, from badly resolved features resulting from a blunt tip to completely different apparent surface structures resulting from multi-tips. Conversely, suitable tips show only one general appearance, that of the expected surface structure with well-defined features, for example on Si(111) - 7 × 7, this would appear as a repeating unit cell containing twelve surface atoms.^1^ We therefore define a binary classification system dividing tip states into “Good” and “Bad” classes. In this way, we can both reduce ambiguity in labeling and increase the accuracy of our machine learning networks. For specific purposes,^22,23^ it may be desirable to distinguish between various desirable tip states, as has been done previously using ML.^14,24^ However, in this paper we focus only on a classifier able to identify a high-quality tip for imaging; therefore, multiple-state classification will not be discussed further. It is important to note that the classification carried out in this study is of the probe tip based on topographical images of the surface rather than the classification of the state of the sample itself, which has been studied previously, for example, by using TEM.^25,26^

## Image Labeling

An essential requirement for the accurate training of ML architectures is a large, well-labeled data set; therefore, a key consideration is to reduce the ambiguity in the training set, which can be achieved by compiling a clear and concise classification scheme that each human labeler is to follow. In general, when labeling a data set with binary labels, such as labeling a set of images of animals “dog” or “not a dog”, it is assumed that the labels being used are completely accurate (i.e., the distinction should be easy to make). However, when labeling a set of images based on an individual’s opinion of the features present, even with a detailed classification scheme, it is inevitable that some ambiguous images remain due to a lack of agreement between labelers. In instances such as these, the images were removed from the classification data set. Images which show a clear tip change mid-image were also removed; an additional script was used to determine whether a tip change had taken place during a scan. Since human classification is itself imprecise, it is important to note that no machine learning classifier would be able to achieve 100% accuracy without significant overfitting.

**Figure 1:**
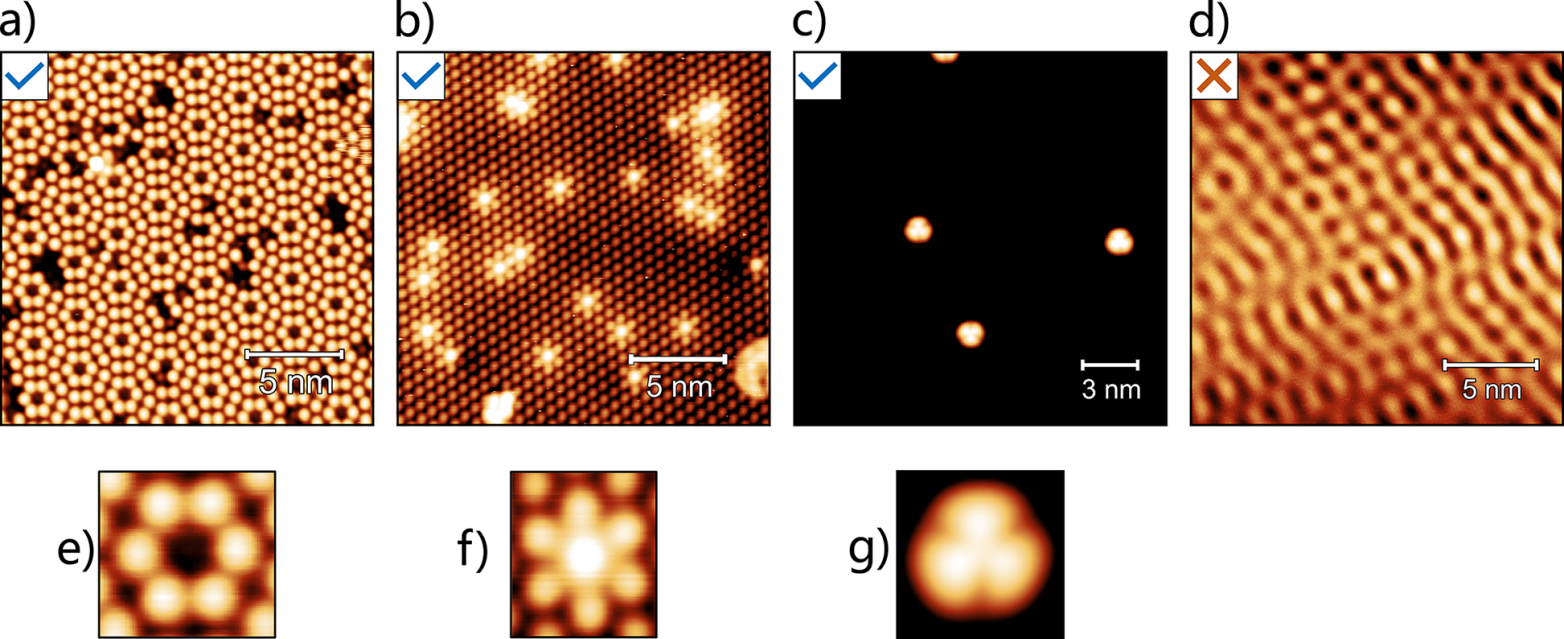
Examples of systems which are (a–c) suitable and (d) not suitable for classification via cross-correlation. (e–g) Example reference images to be used in the CC classification on the surfaces shown in (a–c), respectively. (a) Si(111) - 7 × 7 surface imaged at 2 V and 200 pA. (b) B:Si(111) surface imaged at 2 V and 250 pA. (c) Cu(111) with a low coverage of C_60_ molecules imaged at 5 K, 100 mV, and 100 pA. (d) Bare Cu(111) surface imaged at 5 K, 1 mV, and 1 nA. This surface is not suitable for CC-based classification, as no common repeating features are visible on the surface; only the standing wave pattern of free electrons on the surface is visible. (e) Corner-hole feature with six surrounding silicon surface atoms on the Si(111) - 7 × 7 surface. (f) Dangling-bond feature surrounded by six silicon surface atoms on the B:Si(111) surface. (g) Single C_60_ molecule on the Cu(111) surface.

“Good” and “Bad” tips were defined on a case-by-case basis, depending on the system being studied and the expected appearance of the surface when scanning using a “Good” tip. For the Si(111) - 7 × 7 surface, the main qualifiers of a “Good” tip were the following: the surface adatoms appeared as well-defined with a good contrast between the adatoms and the corner holes, and the appearance of the overall surface was that of the 7 × 7 structure with a diamond-shaped unit cell containing 12 atoms and corner holes at the corners, as shown in Figure 1a. In contrast to this, the B:Si(111) - (√3 × √3)R30° (referred to as B:Si(111) hereafter) surface and the Cu(111) surfaces (Cu(111) with a low coverage of Cu adatoms and CO molecules and Cu(111) with a low coverage of C_60_ molecules) were categorized primarily by the protrusions in the topograph (dangling bonds (DBs) in the case of the B:Si(111) surface and Cu adatoms on Cu(111)), as these features vary a great deal with small changes in the apex of the probe tip. These features appear misshapen when imaged with a “Bad” tip, as opposed to their usual round appearance when imaged with a “Good” tip, and are, hence, highly sensitive tip classification points that allow for clear “inverse imaging” of the probe tip. Finally, on the Cu(111) surface with a low coverage of C_60_ molecules, the molecules themselves are used to classify the state of the probe tip, with a “Good” tip showing the molecules with three lobes (as shown in Figure 1e). These molecules protrude relatively far from the surface (an apparent height of ∼600 pm in STM at 0.1 V and 100 pA with an actual height of ∼1 nm); therefore, the tip state needs to be considered more carefully, as tip defects further up the shaft are more evident during a scan, which can result in the appearance of double/multi-tip features more commonly. Because of this, we only classify the primary apex of the tip rather than the tip as a whole (see the Supporting Information (SI) for further discussion).

The images of the Si(111) - 7 × 7 surface used in this paper were manually labeled by four scanning probe microscopists familiar with atomic-resolution imaging in ultrahigh vacuum (UHV). Initially, the total number of images obtained was 1308, with 873 remaining after the removal of ambiguous scans. Through comparisons between the labelers on this surface, it was found that the overall accuracy of the labeling was not reduced when the data set was labeled by only a single operator. Therefore, the labeling of the other surfaces (specifically the B:Si(111) and one of the Cu(111) surface) was carried out by only a single microscopist (this is discussed further in the SI). Initially, the B:Si(111) data set contained 1701 images, with 1296 remaining after the removal of ambiguous scans or those containing tip changes.

The labeled Cu(111) data set with a low coverage of carbon monoxide molecules and copper adatoms contained a total of 2036 images, with 1996 “Bad” images and 40 “Good” images. In this case, no images were removed, as there was little ambiguity and no tip changes. We note that from the labeling, the data set was highly unbalanced and so would not be amenable to training an ML-based classifier (see the SI for further details). The Cu(111) data set with a low coverage of C_60_ molecules had a similar imbalance in classes and so was not labeled.

In the case of the images labeled by multiple labelers (as carried out on the Si(111) - 7 × 7 surface), ambiguous images were defined as those which the majority of labelers (three out of the four) did not agree, whereas for a single labeler (B:Si(111)), an extra choice of label was included for images that the labeler could not classify with certainty. The collective agreement among all operators was assessed to determine the overall consistency in the labeling. No individual labeler exhibited a deviation from the majority greater than 10% when considering the entire batch of images; this highlights a high level of agreement between the operators.

Additionally, for the Si(111) - 7 × 7 data set, which was labeled by four operators, a random 10% of the images were represented to the labelers to measure each individual’s consistency within their own labeling. Final accuracies and precisions for the operator labels (shown later in Table 1) were calculated based on the most consistent labeler, or in the case of a single labeler, the accuracy and precision were based only on the repeated images. These accuracies were calculated to be used as a comparison between the human labeling, the ML classifier, and the TM classifier and are shown later in the Results and Discussion section.

**Table 1:** Accuracy and TPP of Multiple Tip State Classification Methods: TM Classifier, ML-Based CNN, and Manual Classifications Carried out by an Operator

	TM		CNN		operator	
	Si-7 × 7	B:Si	Si-7 × 7	B:Si	Si-7 × 7	B:Si
accuracy	90%	89%	96%	90%	92%	95%
TPP	97%	95%	92%	97%	93%	88%

## Machine Learning Classifier

A ML-based classifier was trained to be used as a comparison to the TM approach. A CNN-based classifier was chosen, as they are commonly used in image classification tasks due to their ability to extract high-level patterns from the input image. Multiple CNNs were trained in order to find the optimal hyperparameters for our specific data set. This involved training CNNs with varying numbers of convolutional and dense training layers, as well as varying the neurons per layer and the convolutional kernal sizes. The optimal architectures found were the same for both the B:Si(111) and Si(111) - 7 × 7 classifiers. The structure used a total of five 3 × 3 convolutional layers (with 20, 40, 60, 80, and 100 feature maps, respectively) with rectified linear unit (ReLU) activation functions, each separated by 2 × 2 max pooling layers. The convolutional layers were followed by three dense training layers (32, 64, and 128 neurons per layer, in that order) using ReLU activation functions and a final binary output layer using a sigmoid activation function. The training layers were each separated by dropout layers (with probabilities of 0.5, 0.3, and 0.3, respectively) to reduce overfitting. This architecture is described in Figure S8 in the SI. The input to each network consisted of 700 × 700 pixel (19.4 × 19.4 nm^2^) constant-current STM topography images.

To improve the performance of the training, images were augmented further using horizontal and vertical flips as well as 90° rotations, increasing the size of the data set by a multiple of 8. This helps to increase the amount of variance in the training data set, which reduces the level of overfitting during the training process. Overfitting is a process in machine learning where a model learns specific patterns present in the training data, which may not be present in general, and proceeds to use these patterns to make its classifications. This causes the training accuracy to increase at the expense of the accuracy obtained on unseen data. Another method used to reduce overfitting was the inclusion of dropout layers after each training layer. These dropout layers temporarily nullify random neurons in the previous layer, reducing the reliance of the networks on specific neurons in training.

## Template Matching Classifier

The TM classifier was developed in an attempt to work around the largest drawback of using an ML-based classifier: the need for a large labeled data set. The aim of the TM classifier is to have a model that is able to make independent classifications of the state of a scanning probe tip using set algorithms that can be applied to images without the need for any training.

The TM classifier we developed uses standard image analysis techniques, specifically CC and a measure of circularity. We find that these methods are sufficient to classify the state of a probe tip using only a single image when applied to the systems being shown here; we discuss an additional attempted metric in the SI.

### Cross-Correlation

Cross-correlation is a fundamental technique used in image processing and computer vision to analyze the similarity between different parts of an image. It plays a crucial role in tasks such as object recognition, image registration, and feature extraction. By measuring the similarity between two images or by comparing a template with an image, we can identify patterns, locate objects, and align images. At its core, CC involves scanning a reference (or kernel) over an image and computing a similarity measure at each position (known here as the cross-correlation ratio (CCR)).

**Figure 2:**
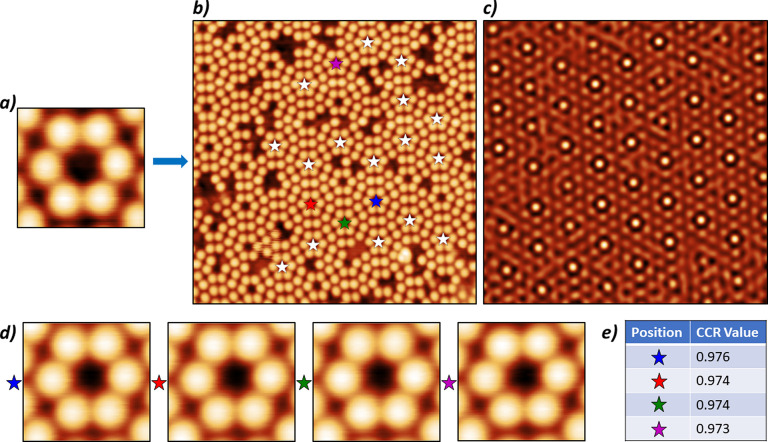
Cross-correlation method as applied to Si(111) - 7 × 7. (a) The reference image used; in this case, the chosen image is a tight square image surrounding a corner-hole feature. (b) An input image over which the reference image will be scanned. Centered over each pixel, the reference image outputs a number between 0 and 1 describing how similar the area is to that of the reference image. The result of this is shown in the cross-correlation feature map in (c). The stars overlaid on (b) show the top 20 highest correlated positions, which correspond to the peaks in (c). (d) Top four highest correlated positions with the colored stars corresponding to the same colored stars in (b). (e) CCR values obtained for the areas shown in (d).

The calculation of the CCR for our use first requires a single small reference image taken from a topograph scan where the scanning probe tip is in an ideal state. The principle of this method is to scan this small section (the reference image) of an ideal image over an input image and measure how closely the reference image resembles the area of the input underneath it at each point. Because of this, care must be taken in choosing the reference image, as a poor choice could lead to inaccurate classifications. The chosen reference image should contain enough information so as to be able to capture a commonly appearing structure, such as a molecule adsorbed on the surface or a unit cell, while being as small as possible. A smaller reference image both reduces the chance of defects in the input image being contained within a highly correlated position and increases the likelihood of multiple instances of the feature being found in the input image. For example, the reference image chosen for the B:Si(111) surface is an image of a single Si DB surrounded by six surface atoms. This structure appears as a bright feature, around which six spheres are arranged in a hexagon. This was chosen for the B:Si(111) surface, as the defect is a common feature and was found to provide higher selectivity for identifying tip quality than the pristine surface. In cases where the tip is not ideal, the surface atoms can appear similar to those when scanning with a “Good” tip, but the DBs often appear misshapen or doubled. In contrast, on the Si(111) - 7 × 7 surface we found the reference image of six atoms surrounding a corner hole was suitable (shown in Figure 2a). Using the chosen reference image, a CC feature map can be calculated using eq 1:^27^
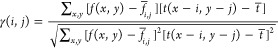
1where γ(*i*, *j*) is the CCR at position (*i*, *j*), *f*(*x*, *y*) is the input image, *t*(*x* – *i*, *y* – *j*) is the reference image at position (*i*, *j*), *t̅* is the mean of the reference image, and *f̅*_*i*,*j*_ is the mean of the area of *f*(*x*, *y*) underneath the reference image at position (*i*, *j*). This was used to generate the feature map, an example of which can be seen in Figure 2c. The reference image is scanned over the input image at every position with the reference centered on each pixel in the input image. The feature map produced will be of the same size as the input image and comprises pixels with values between 0 and 1, with 0 being no correlation at that position and 1 corresponding to an exact match. Bright peaks can be seen in the feature map, corresponding to positions with high input image correlation to the reference image, i.e., areas which appear similar to the center of a corner hole in the example shown (see Figure 2a).

By choosing the top *N* values in the CC feature map and taking an average of these, an overall value for the CCR can be obtained from the input image, which shows overall how closely it resembles the features present in the reference image. The value of *N* here can be chosen depending on how commonly the reference image feature appears in the system in question. Although higher *N* values avoid the chance of a spurious high correlation, for the data sets investigated here, *N* = 1 produced similar results to *N* = 5 and gives the advantage that only one instance of the feature needs to be present in the scan, which could be useful when evaluating surfaces where a high defect density could be present (e.g., in the case of Si(111) - 7 × 7).

Using the technique described above, one obtains a single numeric metric that describes how closely the image matches one taken with an “ideal” tip and can therefore be used as a measure of the quality of the probe tip. For a given sample system, the CCR threshold for a “Good” tip was defined empirically by running a small test set of images (around 20 images would be sufficient) through CC evaluation. Practically, it was found that using a precision of 2 significant figures in the CC value was adequate for high-quality discrimination between tip states using this technique. This process only needs to be performed once for a given sample system and can be easily modified later if it is practically found that the threshold is too strict or too lenient.

### Circularity Measurement

An additional method was developed in order to allow for classifications of the Cu(111) surface using deposited copper adatoms as a comparison point. This method starts by using the same CC algorithm to obtain the highest correlated position of the surface compared to a chosen reference image containing a single adatom. Although this locates adatoms successfully, we found that due to the lack of distinct features within the adatom, we were unable to define a metric using CC that reliably distinguished between “Good” and “Bad” tips. We therefore introduced an additional image classification stage using the measured circularity of adatoms on the surface.

The appearance of the adatoms is highly dependent on the shape of the probe apex, with any irregularities in the tip causing the spherical shape of the adatom to appear deformed. Hence, we find it effective to measure the circularity of the adatoms for use as a metric in our classifier.

Once the adatom has been located on the surface using CC, the image is normalized to be between 0 and 1 and then thresholded to binarize the image, with all values above a specific threshold having a pixel value of 1 and those below having a value of 0. This is repeated for four image thresholds (0.4, 0.5, 0.6, and 0.7), resulting in four output images for each adatom. A range of image thresholds is chosen, as each binarized image corresponds to the shape of the feature at different radii from the center. Thus, by taking an average of a range, it is possible to check how spherical the feature appears. From here, the circularity is measured using eq 2 for each image:
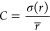
2where σ(*r*) is the standard deviation of the radius and *r̅* is the mean. This results in an output which measures how similar the feature is to a perfect circle, with a perfect circle measuring 0. The Python library PyDIP was used to measure the radius of the feature in each binarized image at various rotations. The average of the four circularity measurements was taken and used as the final metric used in the TM classification. Similar to the CCR metric, an image is classified as “Good” based on a threshold, which is discussed further in Results and Discussion section.

## Results and Discussion

Before discussing the results in detail, we first outline the key metrics for evaluating the classifiers: accuracy and true positive precision (TPP). Accuracy is the simpler of the metrics discussed here and is the percentage of the time that the model is correct in its predictions. Precision, in contrast (specifically TPP), is the proportion of time the model predicts a tip to be “Good” and is correct in that prediction. TPP does not take into account the number of “Good” tips that are incorrectly predicted as “Bad” or in fact any tips classified as “Bad” at all. For the purposes of creating a tip state classifier for an automated tip preparation scheme, this metric is prioritized over accuracy, as it is more essential for this model to be certain in its positive predictions than it is advantageous for it to achieve a high accuracy. A higher accuracy here would contribute to the model taking less time to achieve a “Good” tip classification (as fewer “Good” tips would be considered to be “Bad” and so disregarded), which, while advantageous, is secondary to being certain of a “Good” tip when it identifies one.

### Silicon Surfaces at Room Temperature

A primary objective of this study was to compare the application of ML versus TM classifiers in the classification of a tip state through the interpretation of topographic images. To achieve this, we compared the performance of both classifiers for two prototypical surfaces along with classifications made by human operators.

The values for the accuracy and TPP of the CNN and TM methods (shown in Table 1) were calculated by using a sample set of images that were not included in the CNN training set. The total number of images used for these evaluations was 174 for Si(111) - 7 × 7 and 259 for B:Si(111), both with a roughly 70:30 ratio of “Bad” to “Good” images. For the final TM results, the CCR thresholds used were >0.92 for both surfaces. The specific choice of threshold for the TM metric being used can be selected based on user preference; in our case, we chose to prioritize a high TPP at the slight expense of accuracy, as discussed above. The results for the operator column in Table 1 were calculated as described previously. The total number of images used for this was 130 for Si(111) - 7 × 7 and 170 for B:Si(111) with the same 70:30 ratio of “Bad” to “Good” images.

For the Si(111) - 7 × 7 surface, the final TPP values obtained for the TM- and CNN-based classifiers were 97% and 92% respectively, which shows a slightly higher TPP for the TM classifier. When considering the B:Si(111) results, the final TPPs were 95% and 97% for the TM- and CNN-based classifiers, respectively, showing very similar values. The values obtained for the accuracy, in contrast, show a slightly different trend: for the Si(111) - 7 × 7 surface, final accuracies obtained for the TM- and CNN-based classifiers were 90% and 96% respectively, while for the B:Si(111) surface, the TM- and CNN-based classifiers obtained accuracies of 89% and 90%, respectively. This slightly lower overall accuracy incurred by the TM classifier is almost entirely due to the misclassification of “Good” tips as “Bad” (as is shown by the high TPP) and thus, as mentioned previously, only contributes to slowing the overall process of exiting with a “Good” tip.

Given the similarity in performance between both models, we note the main apparent advantage of using TM image analysis techniques for classification versus ML-based methods is the significantly reduced overhead in their creation. As noted, the sufficiently large data sets that are required for ML are not always available, as was the case for this study, which necessitated the development of an automated data set generation script. In addition to the need for large data sets, manual labeling has to be carried out on these data sets, which is a very time-consuming process and requires careful forethought and trained microscopists to obtain adequate labels. When compared to large labeled data sets used in other fields, it is not possible to outsource the process of labeling a data set, primarily due to the instrumental expertise and physical understanding required to make the distinction between “Good” and “Bad” tips. Conversely, the TM methods require a much smaller data set, comprising a single “Good” sample image, and no time is needed for training. Therefore, when attempting to automate tip state classification on a new system, much less time and effort is needed with very similar overall results being obtained.

Additionally, the results from the presented ML networks show accuracy and TPP values comparable to those of previous attempts published in the literature, as shown in Table 2. The average accuracy and TPP values obtained in previous works were 94% and 93%, respectively, compared to the averages of 93% and 95% obtained in our ML attempts. We note here that direct comparisons between ML networks trained on entirely different data sets are difficult to make due to the variability in the data sets themselves (differences include size, variability of features, and data preprocessing). Therefore, caution should be taken when making quantitative comparisons between the results of different ML-based classifiers. Nevertheless, the results indicate that the ML method presented here is able to classify tip states with high accuracy and TPP, and it performs similarly to other ML SPM image classifiers.^13,14,18^

**Table 2:** Accuracy and TPP of Multiple ML-Based Binary Classification Attempts in the Literature on Various Surfaces^a^

	RW^13^	GM^14^		Krull^18^
	H:Si(100)	H:Si(100)	Au(111)	MgPc/Ag(100)
accuracy	97%	93%	91%	94%
TPP	not given	96%	97%	87%

a The Rashidi–Wolkow (RW),^13^ Gordon–Moriarty (GM),^14^ and Krull^18^ models all use convolutional neural networks.

For the Si(111) - 7 × 7 surface, the operator-based classification resulted in an accuracy of 92% and a TPP of 93%. These results show very similar values when compared to both the TM- and CNN-based classifiers with a standard deviation of 4% in accuracy and 2% in TPP. Similar results were found for the B:Si(111) surface. Additionally, the results from Table 1 support the results from previous works,^13,14^ with the accuracy and TPP values calculated using a traditional CNN being comparable to the results of manual labeling by an operator.

### Adsorbates on Cu(111) at Low Temperature

In addition to the two silicon-based surfaces described above, two data sets were obtained using a Cu(111) surface at 5 K: one with a low coverage of copper adatoms and CO molecules and another with the addition of C_60_ molecules. When imaging high aspect ratio features such as Cu adatoms and C_60_ molecules on Cu(111), imaging is much more sensitive to the shape of the tip, and it is more likely for one to encounter secondary apexes further up the tip shank. As a result, it is more difficult to obtain a tip that is sufficiently sharp to image these high aspect ratio features without observing tip-related artifacts such as “doubled” features. Consequently, this results in a higher proportion of tips being classified as “Bad” on this surface. Due to random tip preparation on these surfaces being less likely to give “Good” tips on these features, the custom LabVIEW script that was used to obtain data sets on the silicon-based surfaces was not as successful at obtaining a balanced data set here. Compared to the roughly 70:30 ratio of “Bad” to “Good” images obtained on the silicon surfaces, the copper surface data sets contained a ratio of 49:1. This hugely unbalanced data set resulted in the training of the CNN-based classifiers failing, even when augmentation strategies were used on the data sets. The specific augmentation strategies used here were horizontal and vertical inversions as well as 90° rotations to artificially increase the size of the data sets. In addition to these augmentations, class weighting was implemented, which should allow the model to adapt to the imbalance; however, the training was still unsuccessful.

**Figure 3:**
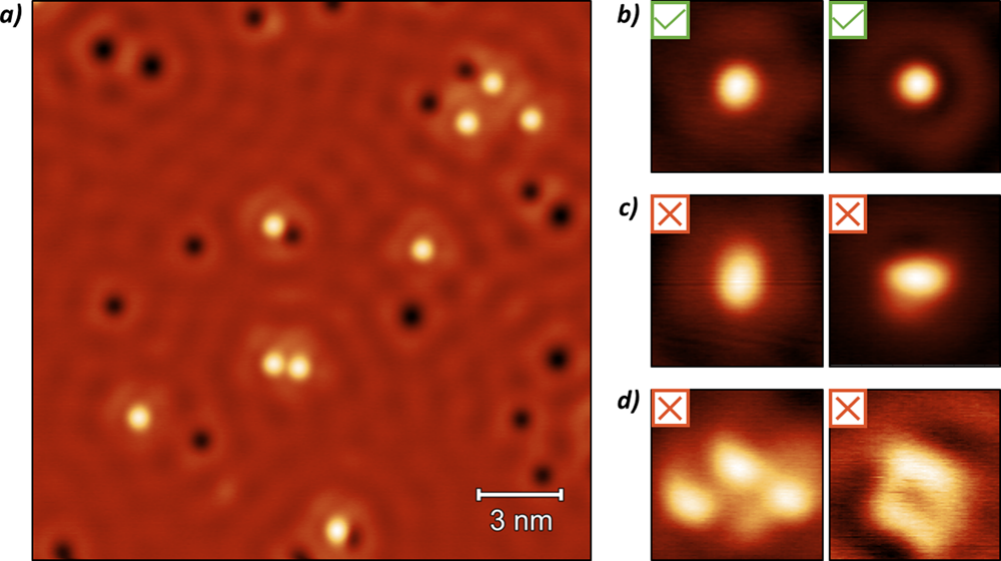
(a) STM image of Cu(111) with a low coverage of Cu adatoms and CO molecules taken at 5 K with an imaging bias of 100 mV and a 100 pA set point. (b) Two examples of Cu adatoms imaged with a “Good” tip, showing a round appearance. (c) Two examples of Cu adatoms imaged with a slightly misshapen tip, which would be classified as “Bad”. (d) Two examples of Cu adatom images attributed to extremely misshapen tips or tips with multiple apexes.

While attempts at classifying the Cu(111) surface using ML were unsuccessful, it was possible to make classifications using TM methods. Here, the adatoms were used to assess the quality of the tip, and an attempt was made to use a single adatom as a reference image to calculate the CCR. However, this method was found to lack sensitivity, and so a different approach was used: the circularity measurement. The reason for the lack of sensitivity is possibly due to the simplicity of the Cu adatom, with small differences in the shape of the circle (such as the oval-shaped adatoms shown in Figure 3c, which were due to slightly misshapen tips) resulting in little change to the CCR.

Using the circularity method with a threshold of <0.035, a final accuracy of 99% and a TPP of 81% were achieved. We note that for highly unbalanced data sets, the accuracy metric is a poor measure, as simply classifying all images as the majority class would result in a high accuracy. Therefore, the TPP provides a much more robust metric by which to assess the performance of the model. For example, the results here were obtained using the entire data set of 2036 images, of which 1996 were classified by a human operator as “Bad” and 40 were classified as “Good”. Because of this, if all images were classified simply as “Bad”, the final accuracy would be 98%.

For the Cu(111) surface with adsorbed C_60_ molecules, it was possible to identify whether the primary apex of the probe tip was “Good” or “Bad” by using CC with a single C_60_ molecule as the reference image. We note that only the primary apex of the tip can be classified, as the height of the molecule can easily result in widely spaced multi-tip features due to tunneling occurring with secondary apexes further up the shaft than is usually considered in imaging. The “shadows” produced by multi-tips are also difficult to identify, as they can often appear similar to the feature shown by the main tip; this is discussed further in the SI. Given the similar imbalance in this data set, when compared to the other copper-based data set, we did not attempt to train this data set with a ML-based classifier and thus did not label all images. Because of this, a final accuracy could not be calculated. However, a TPP was calculated by labeling the images that the CCR method classified as “Good” for a given threshold. It was found that a CCR threshold of >0.99 produced the best results with a TPP of 87% on primary apex identification.

### Automated Tip Preparation Tool

**Figure 4:**
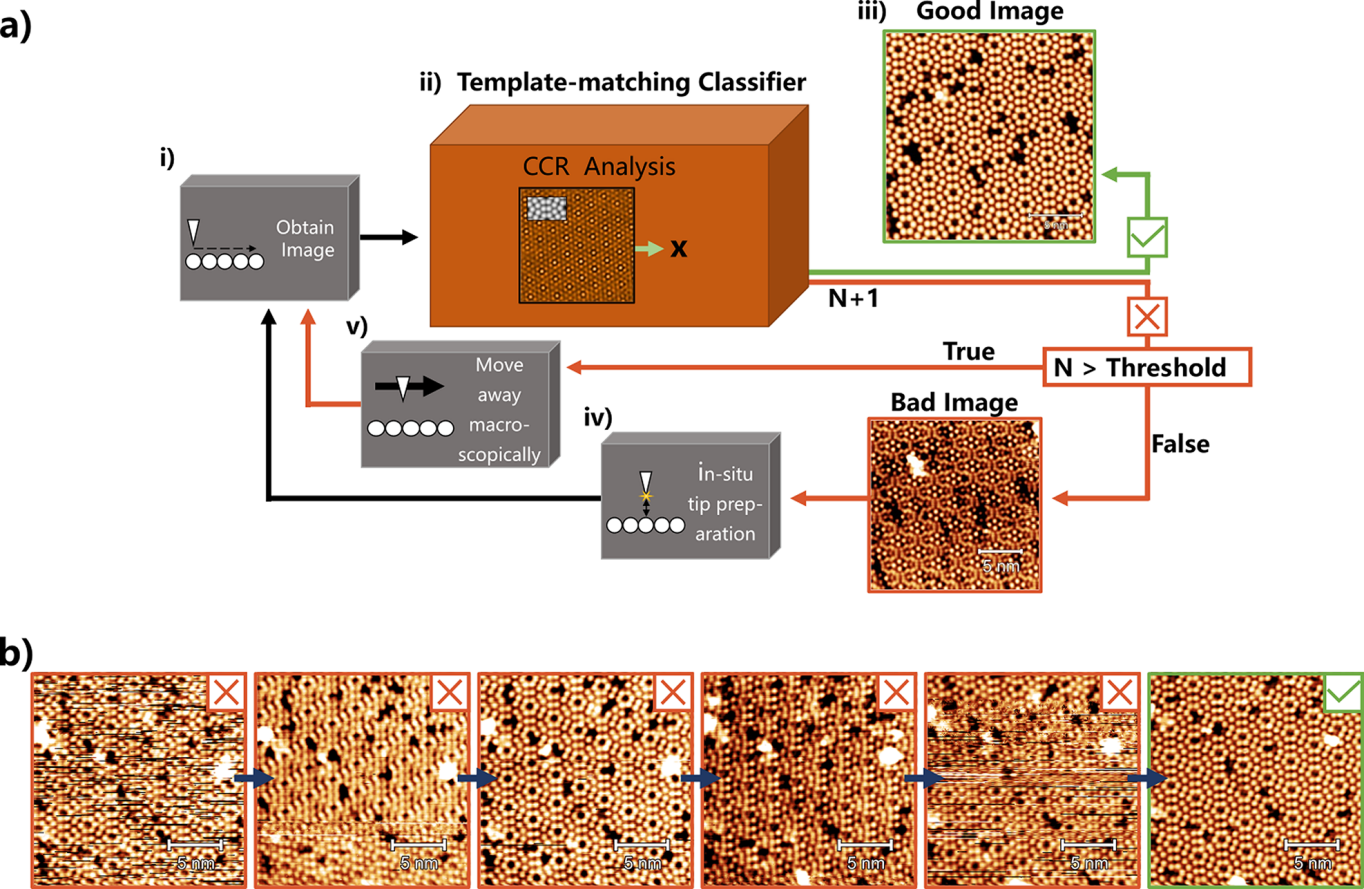
(a) Schematic of the automated tip preparation tool. (i) The process starts by scanning an area to obtain an image of the surface. The image is processed to make it appear flat and to remove the bottom 20 lines from the scan to avoid visible creep before it is then classified. (ii) A cross-correlation-based analysis script compares the input image to a reference and outputs an estimated binary classification of “Good” or “Bad”. (iii) If the tip is classified as “Good”, the script then exits. (iv) If the tip is classified as “Bad”, the script will move away and attempt to reprepare it. (v) If the tip has already been through a set number of shaping events at this point, the script will instead reposition the scan area away macroscopically using the coarse motor, under the assumption that the area being scanned is not suitable to classify the tip. (b) Representative sequence of constant current images showing the Si(111) - 7 × 7 surface at 2 V and 100 pA. The images show the operation of the automated tip preparation tool, which was able to prepare the tip from an initial “Bad” state (far left) to a “Good” tip (far right) in five shaping attempts.

Once a computationally based tip state classification scheme has been produced, it is possible to implement an automated STM tip preparation tool. This tool, similar to the data set generation tool, was implemented in LabVIEW. The tool, schematically shown in Figure 4a, works by repeatedly obtaining topographies of the surface and making classifications of each image. If the topograph is classified as “Bad”, the system moves a predefined distance away from the scan area and attempts to condition the tip in situ. Manual in situ tip preparation involves combinations of two processes: bias pulses applied to the tip, which facilitate the ejection of matter from its surface, and indentations of the tip into the surface in an attempt to refine and sharpen the tip through the detachment or attachment of matter on the tip apex. To emulate this manual procedure, the shaping events themselves were chosen beforehand to increase in magnitude over successive attempts to reduce the chance of getting stuck in a blunt but robust tip state.

The distance the tip needs to move away from the imaging site varies but is usually around 200 nm to ensure the imaging area is not affected by the shaping events, as they can cause the scattering of contaminants in the area. In addition, the script counts the number of attempts taken so far in the preparation, and when the counter exceeds a predetermined threshold, the tip will move macroscopically away from the current scan area by stepping away using the coarse motor. This is done because if the area is damaged or otherwise unsuitable, the automated tip preparation tool will be unable to identify the tip as ever being “Good”.

This tool was implemented using the TM classifier, and proof-of-principle experiments were performed on the Si(111) - 7 × 7 surface. In a trial of 20 runs, the automated tip preparation tool was able to prepare a tip from “Bad” to “Good” after an average of 12 shaping events (full details on a sample set of runs are shown in the SI), which corresponds to approximately 10 min at the scan size and speeds chosen here (scanning a 20 × 20 nm^2^ area with a pixel density of 256 × 256 and a scan speed of 76.8 ms/line). This tool allows for the very time-consuming and tedious process of tip preparation to be completely automated, leaving the user to use their time elsewhere.

An example of a tip preparation run using this tool is shown in Figure 4b, and further example videos are included as online supporting data sets (see Videos S1–S3).

### Applicability and Limitations

The ability of the proposed TM method to make accurate classifications on multiple surfaces using only a single image of the surface being studied makes it a valuable tool; however, there are some limitations which should be noted.

The main metric used is the CCR, which relies on a repeating structure being present on the surface being studied. While this is the case for a very large number of surfaces studied using atomic-resolution STM, as in many of the systems presented here, there are situations where nothing on the surface could be used as a reference image. For example, with the imaging parameters used in Figure 1d, the atomic structure of the Cu(111) surface is not resolved, with only the electron standing wave patterns being visible. These standing wave patterns can vary by a large amount, depending on localized scattering potentials from features such as atomic step edges and adsorbates. We note in passing that it is also difficult for a human operator to assess the quality of the tip from an image of this type.

Conversely, where the appearance of the atomic resolution of the surface changes with bias (e.g., as is the case for positive and negative bias images of Si(111)), the TM model can be “retrained” to assess images at a different bias using only one example image; a ML-based model would require an entirely new training data set and reclassification to adapt. In addition to the need for a repeating structure, the chosen structure needs to contain sufficient detail such that a measurement of the CCR will differ between the tip states. For example, the CCR measurement was not suitable for use on the Cu(111) surface with a low coverage of Cu adatoms and CO molecules (when using a tight crop of either adsorbates as a reference image) due to the fact that the oval-shaped appearance of the adatoms/CO molecules (Figure 3c) caused by slightly misshapen tips still gave high CCR values when compared to a “Good” tip reference image. This resulted in the addition of the circularity measurement metric, which, when combined with feature finding through CC, was able to accurately classify the state of the probe tip. It seems likely, therefore, that a very broad range of atomic- and molecular-scale structures are amenable to classification via a combination of these TM methods.

We also consider the applicability of the TM methods to other surfaces that have previously been classified by ML methods in the literature, in each case commenting on whether the surfaces would suit CC-based methods and, where possible, applying our TM classifier to publicly available data sets.

In the work carried out by Aldritt et al.,^19^ ML was used to classify images of CO molecules to assess the quality of a CO-functionalized tip, which was prepared using an automated tool. Using a CNN, the authors were able to achieve an overall binary accuracy of 95% and a TPP of 90%. When imaging a CO molecule with a “Good” CO-functionalized tip, the molecule appears as a sombrero-like feature with a protrusion centered inside a ring-shaped depression. With this knowledge and by using the publicly available data set used for this work, a “Good” image of this sombrero-like feature was extracted and was chosen as a reference image. This reference image was then used to create a CC-based TM classifier, which was able to achieve an accuracy of 62% and a TPP of 99%. These results show that, while the CC-based method would disregard a larger portion of the “Good” tips compared to the ML-based method, it would be more precise in its final classification of a “Good” tip, which follows the trend we observed in our own data and supports the robustness of the method.

In the work of Krull et al.,^18^ ML was used to classify images of magnesium phthalocyanine (MgPc) on Ag(100) as part of an automated imaging tool. MgPc should be an ideal candidate for classification via CC, as when it is imaged with a “Good” tip, the MgPc molecule has a distinct cross-shaped appearance that would, in theory, allow for the CCR metric to distinguish between tip states. It should be noted that at higher coverages, the appearance of the molecule may change, for example, with the formation of molecular islands. It would therefore be necessary to select a reference image that best reflects the appearance of the molecule on the surface for a given coverage. An attempt was made to classify the open-source data used by Krull et al..^18^ However, the very low resolution of the images in the available online data^18^ for training prevented accurate classification due to a lack of detail in the images.

In the work carried out by Rashidi et al.,^13^ images of DBs on the H:Si(100) surface were used to train an ML-based classifier to determine the state of the probe tip between two states: “sharp” and “double”. The state of the probe tip on the H:Si(100) surface can be characterized in multiple ways, depending on the desired tip mode. The H:Si(100) surface appears as rows, and the state of the tip is classified based on a DB defect in the structure, which appears as a diamond-shaped protrusion. It is possible that the CCR metric would be able to distinguish between a “Good” and “Bad” tip using a cropped image of this defect as a reference image; however, since the aim of their work was to distinguish specifically between “sharp” and “double” tip states, other metrics could be used to make this classification, such as thresholding of the image combined with a way of counting features present in the scan.

There are also cases in the literature in which surfaces have been imaged that would not be suitable to classification via TM methods, such as the work carried out by Gordon et al.^14^ In this work, the authors imaged the Au(111) surface without atomic resolution and in the absence of adsorbates, which showed only the characteristic herringbone structure. Similar to the standing wave pattern visible on the Cu(111) surface, the herringbone structure shows no specific regular features that could be used as a CC reference image, and so, ML-based methods seem necessary.

## Conclusion

A comparison between common machine learning techniques and more traditional image analysis techniques has been presented in the case of scanning tunneling microscope tip state classification using atomic- or molecular-resolution topographical images. We found that using relatively simple image analysis techniques such as cross-correlation produces comparable accuracy and true positive precision values, massively reduces the time needed to create a classification scheme, and drastically reduces the amount of data needed to create a functioning classifier when compared to machine learning methods. Using this method, we were able to classify data sets that were not possible to classify using machine learning, and we also applied our methodologies to publicly available experimental data sets, obtaining comparable classification precision results. This suggests that the TM classifier is a robust, easily implemented, and widely applicable methodology for atomic-resolution studies.

In addition, an automated tip preparation tool was implemented using the TM classifier, which is able to successfully obtain and maintain a stable probe tip, highlighting the proof-of-principle application of this technique in the automation of scanning tunneling microscopy experiments.

We note that scripts such as this could be included in any automated experiment script that involves periodically scanning the surface, meaning it is applicable for both obtaining a usable tip to start an experiment and maintaining it throughout random tip change events. An automated script such as this could be implemented alongside the autonomous manipulation of individual atoms and molecules on a surface for device fabrication or setting up precise experiments.^28,29^

We have shown that TM techniques are able to achieve comparable accuracies and precisions in the case of tip state classification when compared to frequently used machine learning-based classifiers. As noted above, there remain instances in which machine learning is a more suitable choice, and thus, it remains essential to consider the problem at hand when making a choice of what computational tool to use.

## Methods

### Experimental Methods

All topographical images used for training were acquired in constant-current mode with a scan frame of size 20 × 20 nm^2^ and a resolution of 720 × 720 pixels. Si(111) - 7 × 7 scans were obtained using a tunnel current set point of 200 pA and a bias voltage of 2 V. B:Si(111) scans were obtained using a tunnel current set point of 250 pA and a bias voltage of 2 V. Cu(111) scans were obtained using a tunnel current set point of 100 pA and a bias voltage of 100 mV.

Room-temperature data were acquired using a commercial Omicron NanoTechnology VT-STM/AFM instrument that was operated using an RC5 Nanonis controller, with all experiments being carried out under ultrahigh vacuum (UHV) conditions. Clean Si(111) - 7 × 7 surfaces were prepared by flash annealing an n-type Si(111) wafer (0.001–0.005 Ω cm) at ∼1200 °C, cooling them to ∼900 °C quickly, and then slowly cooling them to room temperature over a period of a few minutes while maintaining a pressure of <2 × 10^–9^ mbar. A clean B:Si(111) - (√3 × √3)R30° surface was prepared via flash annealing heavily boron-doped Si(111) wafers (0.001–0.005 Ω cm) to ∼1200 °C for 10 s before quickly cooling them to ∼800 °C and annealing them at ∼800 °C for 1 h. This was followed by cooling the surface slowly to room temperature over a period of roughly 20 min, maintaining a pressure of <3 × 10^–9^ mbar throughout. Low-temperature data were acquired using a commercial Omicron NanoTechnology LT-STM instrument that was operated using an RC5 Nanonis controller, with all experiments being carried out under UHV conditions. Clean Cu(111) surfaces were prepared by standard sputter–annealing cycles with a beam energy of 1.5 keV and an annealing temperature of 500 °C. A low coverage of Cu and C_60_ was deposited on the Cu(111) surface by direct sublimation into the scan head from a FOCUS EFM 3T evaporator, during which the sample was held at ∼5 K. A low coverage of CO on Cu(111) was achieved by leaking CO (to a pressure of around 10^–8^ mbar) into the chamber for 30 s while the sample was kept below 10 K. All images on the Cu(111) surface were recorded at ∼5 K. Electrochemically etched tungsten STM tips were used with the addition of cleaning prior to imaging via electron bombardment for the room-temperature data. Small alterations to the tip were made in situ via standard STM techniques.

### Computational Methods

Scripts for automated image acquisition and the tip preparation tool were created by using LabVIEW and interfaced directly with the Nanonis controller. Scripts for training the machine learning network and the TM classification were written in Python. For the ML training, the TensorFlow package was used. PyTorch was also explored, and a comparison between the two packages was performed; however, it was found that there was no significant difference between the two packages for the data sets analyzed in this paper.

## Supporting Information Available

Video S1: example of a tip preparation run using the automated tip preparation tool (MP4)
Video S2: another example of a tip preparation run using the automated tip preparation tool (MP4)
Video S3: another example of a tip preparation run using the automated tip preparation tool (MP4)
The image acquisition process, a Fourier ring correlation discussion, a data sets discussion, the labeling process, the tip preparation tool, specifics of imaging on different substrates in STM, and additional machine learning details (PDF)

## References

[ref1] Binnig G.; Rohrer H.; Gerber C.; Weibel E. 7 × 7 Reconstruction on Si(111) Resolved in Real Space. Phys. Rev. Lett. 1983, 50, 120–123. 10.1103/PhysRevLett.50.120.

[ref2] Sugimoto Y.; Pou P.; Abe M.; Jelinek P.; Pérez R.; Morita S.; Custance Ó. Chemical identification of individual surface atoms by atomic force microscopy. Nature 2006 446:7131 2007, 446, 64–67. 10.1038/nature05530. 17330040

[ref3] Gross L.; Mohn F.; Moll N.; Liljeroth P.; Meyer G. The chemical structure of a molecule resolved by atomic force microscopy. Science 2009, 325, 1110–1114. 10.1126/science.1176210. 19713523

[ref4] Stroscio J. A.; Celotta R. J. Controlling the Dynamics of a Single Atom in Lateral Atom Manipulation. Science 2004, 306, 242–247. 10.1126/science.1102370. 15358867

[ref5] Ternes M.; Lutz C. P.; Hirjibehedin C. F.; Giessibl F. J.; Heinrich A. J. The force needed to move an atom on a surface. Science 2008, 319, 1066–1069. 10.1126/science.1150288. 18292336

[ref6] Kolmer M.; Godlewski S.; Lis J.; Such B.; Kantorovich L.; Szymonski M. Construction of atomic-scale logic gates on a surface of hydrogen passivated germanium. Microelectron. Eng. 2013, 109, 262–265. 10.1016/j.mee.2013.03.061.

[ref7] Fuechsle M.; Miwa J. A.; Mahapatra S.; Ryu H.; Lee S.; Warschkow O.; Hollenberg L. C.; Klimeck G.; Simmons M. Y. A single-atom transistor. Nat. Nanotechnol. 2012, 7, 242–246. 10.1038/nnano.2012.21. 22343383

[ref8] Leinen P.; Esders M.; Schutt K. T.; Wagner C.; Muller K.-R.; Tautz F. S. Autonomous robotic nanofabrication with reinforcement learning. Science Advances 2020, 6, eabb6987 10.1126/sciadv.abb6987. 32917594 PMC7467688

[ref9] Welker J.; Weymouth A. J.; Giessibl F. J. The influence of chemical bonding configuration on atomic identification by force spectroscopy. ACS Nano 2013, 7, 7377–7382. 10.1021/nn403106v. 23841516

[ref10] Herz M.; Giessibl J.; Mannhart J. Probing the shape of atoms in real space. Phys. Rev. B 2003, 68, 045301 10.1103/PhysRevB.68.045301.

[ref11] Silver D.; et al. Mastering the game of Go without human knowledge. Nature 2017, 550, 354–359. 10.1038/nature24270. 29052630

[ref12] Floridi L.; Chiriatti M. GPT-3: Its Nature, Scope, Limits, and Consequences. Minds and Machines 2020, 30, 681–694. 10.1007/s11023-020-09548-1.

[ref13] Rashidi M.; Wolkow R. A. Autonomous Scanning Probe Microscopy in-situ Tip Conditioning through. ACS Nano 2018, 12, 5185–5189. 10.1021/acsnano.8b02208. 29790333

[ref14] Gordon O.; D’Hondt P.; Knijff L.; Freeney S.; Junqueira F.; Moriarty P.; Swart I. Scanning Probe State Recognition With Multi-Class Neural Network Ensembles. Rev. Sci. Instrum. 2019, 90, 103704 10.1063/1.5099590.

[ref15] Gordon O.; Junqueira F.; Moriarty P. Embedding Human Heuristics in Machine-Learning-Enabled Probe Microscopy. Machine Learning: Science and Technology 2020, 1, 015001 10.1088/2632-2153/ab42ec.

[ref16] Wang S.; Zhu J.; Blackwell R.; Fischer F. R. Automated tip conditioning for scanning tunneling spectroscopy. J. Phys. Chem. A 2021, 125, 1384–1390. 10.1021/acs.jpca.0c10731. 33560124

[ref17] Rashidi M.; Croshaw J.; Mastel K.; Tamura M.; Hosseinzadeh H.; Wolkow R. A. Deep learning-guided surface characterization for autonomous hydrogen lithography. Machine Learning: Science and Technology 2020, 1, 025001 10.1088/2632-2153/ab6d5e.

[ref18] Krull A.; Hirsch P.; Rother C.; Schiffrin A.; Krull C. Artificial-intelligence-driven scanning probe microscopy. Communications Physics 2020, 3, 54 10.1038/s42005-020-0317-3.

[ref19] Alldritt B.; Urtev F.; Oinonen N.; Aapro M.; Kannala J.; Liljeroth P.; Foster A. S. Automated tip functionalization via machine learning in scanning probe microscopy. Comput. Phys. Commun. 2022, 273, 108258 10.1016/j.cpc.2021.108258.

[ref20] Woolley R. A.; Stirling J.; Radocea A.; Krasnogor N.; Moriarty P. Automated probe microscopy via evolutionary optimization at the atomic scale. Appl. Phys. Lett. 2011, 98, 253104 10.1063/1.3600662.

[ref21] Stirling J. Scanning Probe Microscopy from the Perspective of the Sensor. Ph.D. Dissertation, University of Nottingham, 2014.

[ref22] Sugimoto Y.; Yurtsever A.; Abe M.; Morita S.; Ondráček M.; Pou P.; Pérez R.; Jelínek P. Role of tip chemical reactivity on atom manipulation process in dynamic force microscopy. ACS Nano 2013, 7, 7370–7376. 10.1021/nn403097p. 23906095

[ref23] Sugimoto Y.; Miki K.; Abe M.; Morita S. Statistics of lateral atom manipulation by atomic force microscopy at room temperature. Physical Review B - Condensed Matter and Materials Physics 2008, 78, 205305 10.1103/PhysRevB.78.205305.

[ref24] Zhuo D.; Hou L.; Masayuki A. Probe conditioning via convolution neural network for scanning probe microscopy automation Hybrid strategies in nanolithography. Applied Physics Express 2023, 16 (8), 085002 10.35848/1882-0786/acecd6.

[ref25] Ziatdinov M.; Maksov A.; Kalinin S. V. Learning surface molecular structures via machine vision. npj Computational Materials 2017, 3 (1), 31 10.1038/s41524-017-0038-7.

[ref26] Ziatdinov M.; Dyck O.; Maksov A.; Li X.; Sang X.; Xiao K.; Unocic R. R.; Vasudevan R.; Jesse S.; Kalinin S. V. Deep Learning of Atomically Resolved Scanning Transmission Electron Microscopy Images: Chemical Identification and Tracking Local Transformations. ACS Nano 2017, 11, 12742–12752. 10.1021/acsnano.7b07504. 29215876

[ref27] Lewis J. P. Fast Template Matching. Vis. Interface 1994, 95, 120–123.

[ref28] Celotta R. J.; Balakirsky S. B.; Fein A. P.; Hess F. M.; Rutter G. M.; Stroscio J. A. Invited Article: Autonomous assembly of atomically perfect nanostructures using a scanning tunneling microscope. Rev. Sci. Instrum. 2014, 85 (12), 121301 10.1063/1.4902536. 25554264

[ref29] Kalff F. E.; Rebergen M. P.; Fahrenfort E.; Girovsky J.; Toskovic R.; Lado J. L.; Fernández-Rossier J.; Otte A. F. A kilobyte rewritable atomic memory. Nat. Nanotechnol. 2016, 11, 926–929. 10.1038/nnano.2016.131. 27428273

