# Supplementary material for: Automated Scanning Probe Tip State Classification without Machine Learning
Source: ACS Nano. 2024 Jan 9;18(3):2384–94. doi: 10.1021/acsnano.3c10597 (PMC10811750; doi:10.1021/acsnano.3c10597)
Supplement: Supplementary file 4 — nn3c10597_si_004.pdf [file nn3c10597_si_004.pdf]

Automated Scanning Probe Tip State Classification Without Machine Learning: Supplementary Information

Dylan Stewart Barker,* Philip James Blowey, Timothy Brown, and Adam Sweetman*

The School of Physics and Astronomy, Bragg Centre for Materials Research, The University of Leeds, Leeds, LS2 9JT, United Kingdom

E-mail: pydsb@leeds.ac.uk; a.m.sweetman@leeds.ac.uk

Image Acquisition

For the purposes of dataset generation and evaluation of the different methods, we selected a standardised size, and parameter set for each surface used.

For the purposes of training the ML model and evaluation of both the template-matching (TM) and ML performance, large training datasets were generated using automated scripting. To this end, a custom LabVIEW script was used, interfacing with the Nanonis SPM controller, which aimed to scan the same imaging area, whilst applying tip preparation steps between subsequent scans (at least 50 nm away from the imaging site) in order to create random tip variations. The tip preparation steps used here involved indenting the tip into the surface, sometimes combined with a voltage pulse being applied to the tip whilst lifting. In this way, the script was able to produce a large number of images with varying tip states without the need for constant operator monitoring.

When acquiring data for surfaces at room temperature, it is important to address the issue of thermal drift, which can result in significant distortions to the apparent surface structure.[1,2] One method to compensate this drift is by using atom tracking,[3] which works by locking the tip on to a surface feature and tracking its displacement over time and hence apply a feed-forward correction vector to the piezo scan tube to compensate. This compensation needs to be regularly updated as the drift between the tip and sample varies over time. However, this method is less suitable for an automated script which deliberately induces large tip changes, as the initial tracking position needs to be manually chosen for the displacement to be measured. To overcome this issue, the image acquisition script also includes a cross-correlation (CC) based drift detector, which identifies the physical drift between two consecutive images and updates the drift vector. In this way, the drift compensation can be updated between successive images. Maintaining the same scan region also reduces the chance of the tip drifting over an area which will produce less suitable images, such as a step edge or disordered area of the surface. We therefore note that all data considered in this paper were acquired in the absence of significant distortion due to thermal drift.

Prior to use in labelling, training, and evaluating, the obtained images underwent minor augmentations which prepare the image as would be done in normal processing by a human operator. These augmentations include plane flattening the images (to reduce the effect of a sample misalignment on the appearance of the topograph), median subtraction by row, thresholding, and removing 20 scan lines from the bottom of the image to remove any visible creep artifacts caused by the prolonged relaxation of the piezoelectric scanner after moving large distances for tip treatment. After these augmentations, the final images were 700×700 pixels (19.4×19.4 nm^2).

Effect of Tips on Imaging (Extended)

Due to the nature of STM operation, the shape of the probe tip plays a significant role in the interpretation of topographical images. Scans of topographical features which have a larger aspect ratio than the tip are not always representative of the feature itself. This is because the image acquired is a convolution of both the shape of the feature, and the shape of the tip. This means that a sharp feature can only be imaged accurately with an equally sharp (or sharper) tip. Figure S1 shows multiple examples of topographical images of the Si(111) - 7×7 surface, taken over the same area, with changes being made to the tip between each.

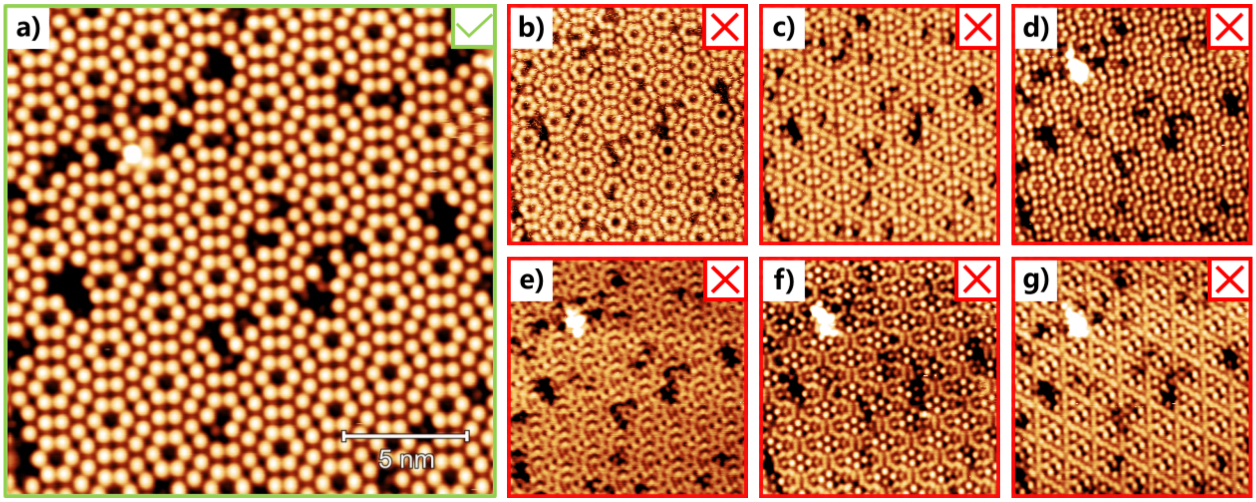

Figure S1: Constant current STM images of the same region of a clean Si(111) - 7×7 surface taken at 2 V, 200 pA. a) was imaged using a tip which would be considered as "Good", whereas images b)-g) would be considered "Bad" and taken with a misshapen tip.

Fourier Ring Correlation

An additional technique attempted to classify the state of the probe tip was Fourier ring correlation (FRC),[4] a method which aims to estimate the spatial resolution of an image. Typically, resolution would be estimated by measuring the minimum resolvable distance between two features, however this requires manual input in choosing and measuring multiple features in the same image to improve the estimate. FRC has been used also[5] to estimate

image resolution in electron cryomicroscopy and optical nanoscopy, however the method is applicable to a wide range of methods including scanning probe microscopy (SPM). The method is based on calculating a cross-correlation histogram in frequency space of two images of the same subject area with independent noise realisations. The spatial frequency spectra of two images are split into bins of different frequencies, which in 2D frequency space corresponds to a series of concentric rings with frequency corresponding to the radius of the ring. The FRC histogram for each frequency bin is calculated using Equation 1.[5]

$$FRC(r_i) = \frac{\sum_{r \in r_i} F_1(r) \cdot F_2(r)^*}{\sqrt{\sum_{r \in r_i} F_1^2(r) \cdot \sum_{r \in r_i} F_2^2(r)}} \tag{1}$$

Where F_1 and F_2 are the Fourier transforms of two images and r_i is the ith frequency bin. The image resolution is defined from the obtained histogram as the inverse of the spatial frequency at which the cross-correlation curve drops below a pre-determined threshold value. In practise, the threshold value used can vary, however we found that the commonly used 1/7 (0.143) threshold[5] works well in predicting a resolution which correspond well to rough qualitative analysis of the images.

Originally, FRC analysis would use two images of the same area with independent noise realizations, however in this use case it is not viable. Koho *et al*[5] introduced a method of calculating the FRC resolution from a single image. This method involves first splitting the input image into two pairs, shown in Figure S2a). The first pair is formed by taking every pixel with (even, even) and (odd, odd) row/column indexes, with the second pair formed from (even, odd) and (odd, even) indexes. The resultant sub-images will appear almost identical, with a size exactly half that of the input image. The FRC can then be calculated for both pairs of sub-images, and an average resolution calculated for the final FRC value of the image.

As can be seen in Figure S3, a weak correlation can be seen between the FRC estimated resolution and the quality of the probe tip, with no "Good" tips appearing below an FRC resolution of around 120 pm. However, within this boundary of images below 120 pm,

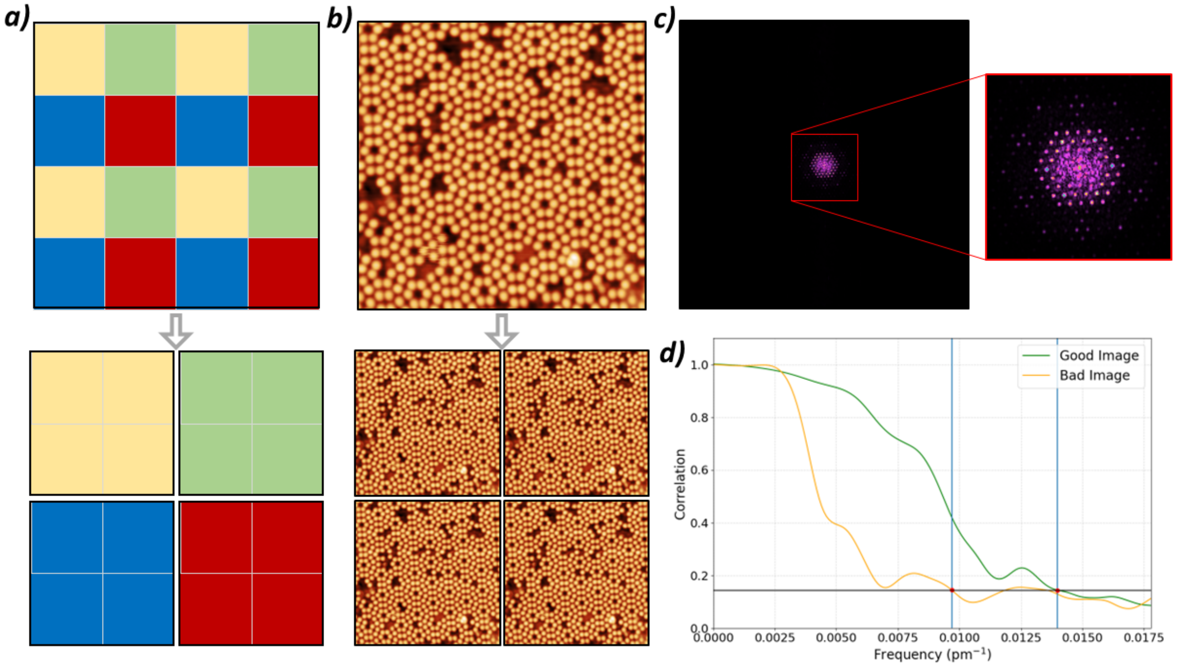

Figure S2: The process of estimating the resolution of an image using FRC. a) First the input image is split into four sub-images by taking every other pixel on each axis. An example of this process, applied to a 4 x 4 pixel image of coloured squares, is shown. b) Shows the input image for a Si(111) - 7 $\times$ 7 sample with the 4 sub-images it is split into. For each of the sub-images, the Fourier transform of the image is obtained, an example of which is shown in c). The inset of c) shows a zoomed in section of the central portion where most of the detail is contained. From here, the cross-correlation is calculated between rings of frequencies in Fourier space between two of the sub images, this produces the plot shown in d). The FRC resolution is determined as the reciprocal of the point (vertical blue lines) at which the resolution drops below a pre-defined threshold (horizontal black line), here we use the commonly used 1/7 threshold. The green line in d) shows the FRC curve of a good image, and the amber shows a bad image.

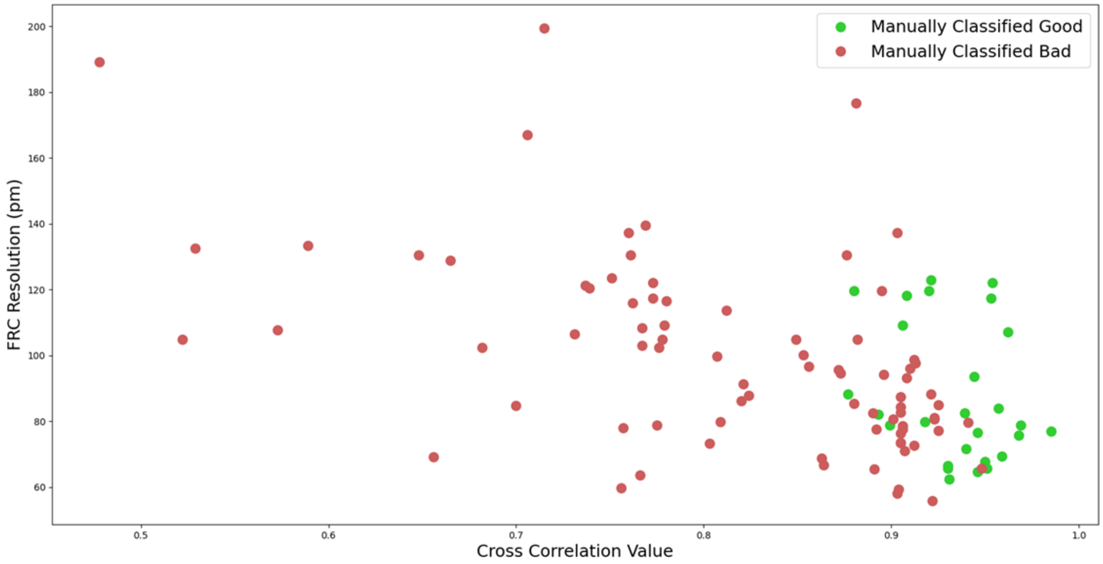

Figure S3: Scatter plot relating the FRC resolution (y-axis) and CCR (x-axis) for various images taken with “Good” (green) and “Bad” (red) tips.

numerous “Bad” tips also appear. FRC was therefore discarded as a metric, as it offered worse selectivity than cross-correlation ratio (CCR). This is to be expected, as in SPM, a good spatial resolution is not always a good descriptor of a “Good” tip, specifically in the case of a multi-tip. It is possible for a multi-tip to have multiple sharp apexes, meaning the resolution of the image will appear to be very good, however the structure in the scan will not be representative of the true atomic or molecular structure of the sample.

Datasets

Many groups[6–8] have access to large historical datasets from previous experiments. Using these for training a ML network simplifies the process of obtaining a suitable dataset, as only the (still non-trivial) process of cleaning and labelling the data is required. Contrary to this, our group did not have access to any prior datasets (which we note would be the case for any group starting investigation of a new sample system). Consequently, this resulted

in the need for an efficient method of obtaining large datasets to be implemented, which is discussed in the main text.

An important note regarding the automated image acquisition script is that whilst it performed well on the silicon based surfaces (Si(111) - 7×7 and B:Si(111) - $(\sqrt{3} \times \sqrt{3})$R30°) in that it was possible set up the script, leave it running overnight and obtain a large, well balanced dataset, this was not the case for either the Cu(111) with Cu adatoms, or the Cu(111) with C_{60} systems (see the main text for a discussion of the quality of the Cu(111) dataset and ML attempts). This lack of "Good" tips is likely to be due to the specific features being used for the classification of the tip state (Cu adatoms and C_{60} molecules) having a high aspect ratio, which results on the features being more sensitive to minor tip distortions when compared to "in plane" features being used on the silicon based surfaces.

While it is possible that longer runs, or modifying the tip preparation parameters may have eventually resulted in a more balanced dataset suitable for ML, we rather highlight the limitation of ML techniques in these scenarios, and show the relative benefit of TM methods under the circumstances of unbalanced or otherwise poor training datasets. For this reason we chose not to put more time into data gathering on the Cu(111) surface to achieve a usable ML dataset, rather we attempted classification of the unbalanced dataset using only ML methods to show their applicability.

Labelling

Initially, we planned for all datasets to be manually labelled by four human operators who were each familiar with the systems being used. However, after the first dataset (Si(111) - 7×7) was labelled in this manner, it was found that taking a majority vote of labels between four operators had little effect on either the training set or the final trained model when compared to using the labels from a single individual. It was found that on average each labeller agreed with the final majority vote 94% of the time. The majority voted labels were also directly compared to the individually labelled set by using both to build a TM

Table S1: Table comparing the results of two TM classifiers trained on labels obtained from the majority vote of four human operators and labelling carried out by a single individual.

	Majority Voting	Solo Labelling
Accuracy	90.2%	90.8%
TPP	94.6%	97.3%

classifier, where the final accuracies and TPPs achieved were almost identical (as shown in table S1). Therefore, the majority voted labels were used for the Si(111) - 7×7 surface, with all other surfaces being labelled by a single operator.

Drift Compensation

As briefly mentioned in the main text, a cross correlation (CC) based drift detector was implemented in order to maintain the position of the scan frame and reduce the chance of the frame moving over a step or disordered region. This drift detector works by taking the CC of two consecutive images, and assuming the highest correlation position corresponds to the position that the centre of the first image has drifted to after the second image. Also, by taking the time between successive scans the drift in m/s can be calculated and updated in the scanning software to apply an appropriate feed-forward vector, which compensates the residual thermal drift during the period of the next scan.

We found the CC based drift detector is able to detect the residual drift accurately even after a tip change has occurred, this is possible as the CC algorithm will find the drift based on features which are still present in both images such as defects and adsorbates, even if the exact contrast on the atoms has changed. To avoid applying erroneous drift compensation, the drift detector outputs an additional error metric, which describes the translation invariant normalised RMS error between the reference and input image. It was found that by disregarding drift vectors with an error value of more than 0.2, erroneous updates could be avoided.

Imaging C_{60}

Due to the large apparent height of the C_{60} molecule (approximately 0.6 nm at 0.1 V, 100 pA), compared to other adsorbates such as adatoms (Cu appears as a protrusion of roughly 40 pm at the same scan parameters) and CO molecules (which appear as depressions around 10 pm in depth), when imaging a system with C_{60} on the surface, it is much more common for portions of the tip further away from the surface to affect the current. Subsequently, shadows or "ghost" features of C_{60} molecules are very common. An example of these shadows are shown in Figure S4, where a) shows four C_{60} molecules when imaging with a "Good" tip and b) shows the subsequent image after a tip change has occurred where additional features with the characteristic appearance of C_{60} but a smaller apparent height can be seen. The new features share the same lateral spacing as three of the molecules from the original image, indicating that they are likely the shadows resulting from a double-tip.

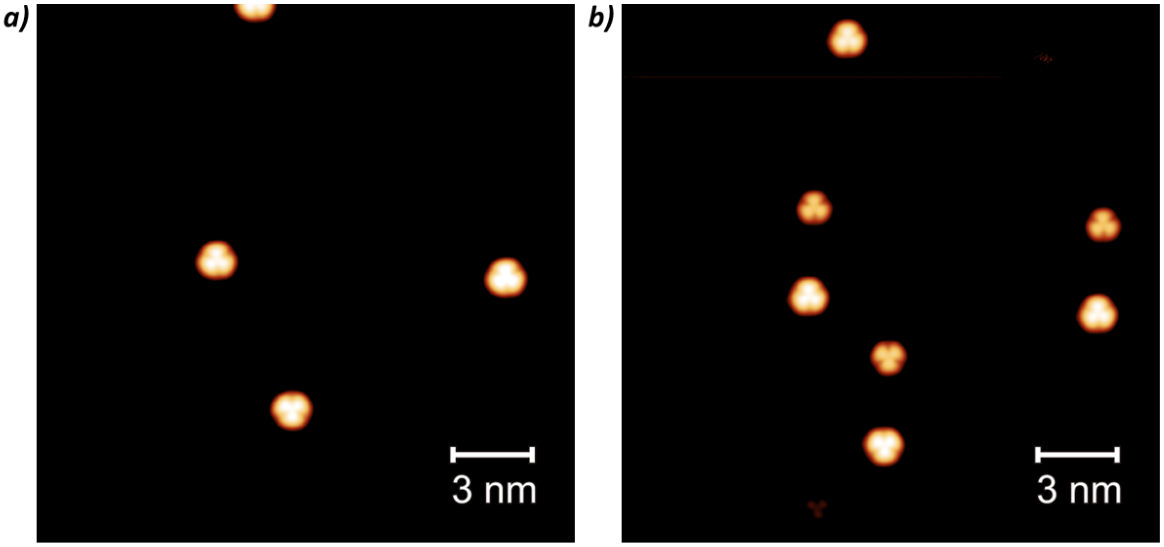

Figure S4: STM scans of the Cu(111) surface with a low coverage of C_{60}. The image was taken at 100 mV imaging bias and 100 pA setpoint. a) and b) show the same imaging area before, a), and after, b), a tip change, where "ghost" C_{60} molecules with a lower apparent height appear.

This appearance of shadows poses a problem for the C_{60} on Cu(111) dataset. Because the criteria (in terms of the required aspect ratio of the tip which would produce an image without any additional artefacts) is so strict, there were a number of tips which produced "Good" imaging of the individual molecules, but with shadows a large distance away. A very small number of tips produce "Good" imaging of the molecules without any long distance artefacts. Therefore, in our analysis, the few tips which would usually appear as "Good" on the Cu(111) surface may show shadows when scanning a surface containing C_{60} molecules, as can be seen in Figure S4b). For this reason when classifying the state of the tip on this system it was decided to classify the quality of the primary tip. This is because although CC is "Good" at finding desired features, it is more difficult (although not impossible), to select and identify a range of "undesireable" features in the same image. The CC based classification for this system is based on using a single cropped images of a C_{60} molecule images with a sharp, metallic tip as a reference image and applying the same method as described in the main text. Using this, both images shown in Figure S4 would achieve a "Good" primary tip classification due to the presence of ideally shaped C_{60} molecules at the correct height, regardless of the shadows also being present.

Cu(111) Machine Learning Attempts

Once the Cu/CO low coverage on Cu(111) dataset was gathered and labelled, it became apparent that the dataset was heavily biased toward "Bad" tips with a total of 1996 "Bad" and 40 "Good" tips. Despite this uneven dataset, attempts to train a ML network were made, with some additions in order to attempt to account for this.

Initial attempts were made with the dataset as it was. However, as expected, the model experienced significant overfitting, learning to classify the majority of images as "Bad" as this would still result in a high accuracy but only achieved a true positive precision of around 20%. Multiple runs were attempted where the number of "Bad" labelled images in

the dataset was reduced in order to achieve a more balanced dataset. For this, the aimed split was 70:30 in favour of the "Bad" tip state to replicate the balance achieved in the working silicon surface datasets, which resulted in a total of 86 "Bad" images and the same 40 "Good" images. With this small training set the model was unable to train well achieving a maximum accuracy of 75% with a maximum precision of around 55%. We attribute the poor performance to an insufficient amount of data in the training set.

In an attempt to improve the performance of the ML classifier, weights were applied to each class. This weighting gives the classes with the smaller amount of images (such as the "Good" tip class) more of an impact on the loss whilst training. The loss is a measure of how far the classifier is away from making a correct prediction on average. Therefore, by weighting the "Good" tip class higher, the amount of overfitting should be drastically reduced when using the whole dataset. The results of two of these training runs are shown in Figure S5, where the maximum accuracy and precision achieved was around 80% and 60% respectively.

As can be seen in Figure S5 by comparing the training runs of a working dataset (c-d) with the Cu(111) dataset, when the training is working well, the precision should increase to a reasonably high value within only 50 - 100 epochs, whereas in the case of the Cu(111) dataset this is not the case.

Si(111) - $7{\times}7$ and B:Si(111) Machine Learning Classifiers

Both of the ML based CNN models trained on the Si(111) - 7×7 and B:Si(111) surfaces (discussed in the main text) were trained using the same model architecture, which can be seen in Figure S8.

Additional testing was carried out on the trained models, to determine the overall performance, by calculating the receiver operator characteristic (ROC) curve. The ROC curve is calculated by making a number of predictions (174 images for the Si(111) - 7×7 model

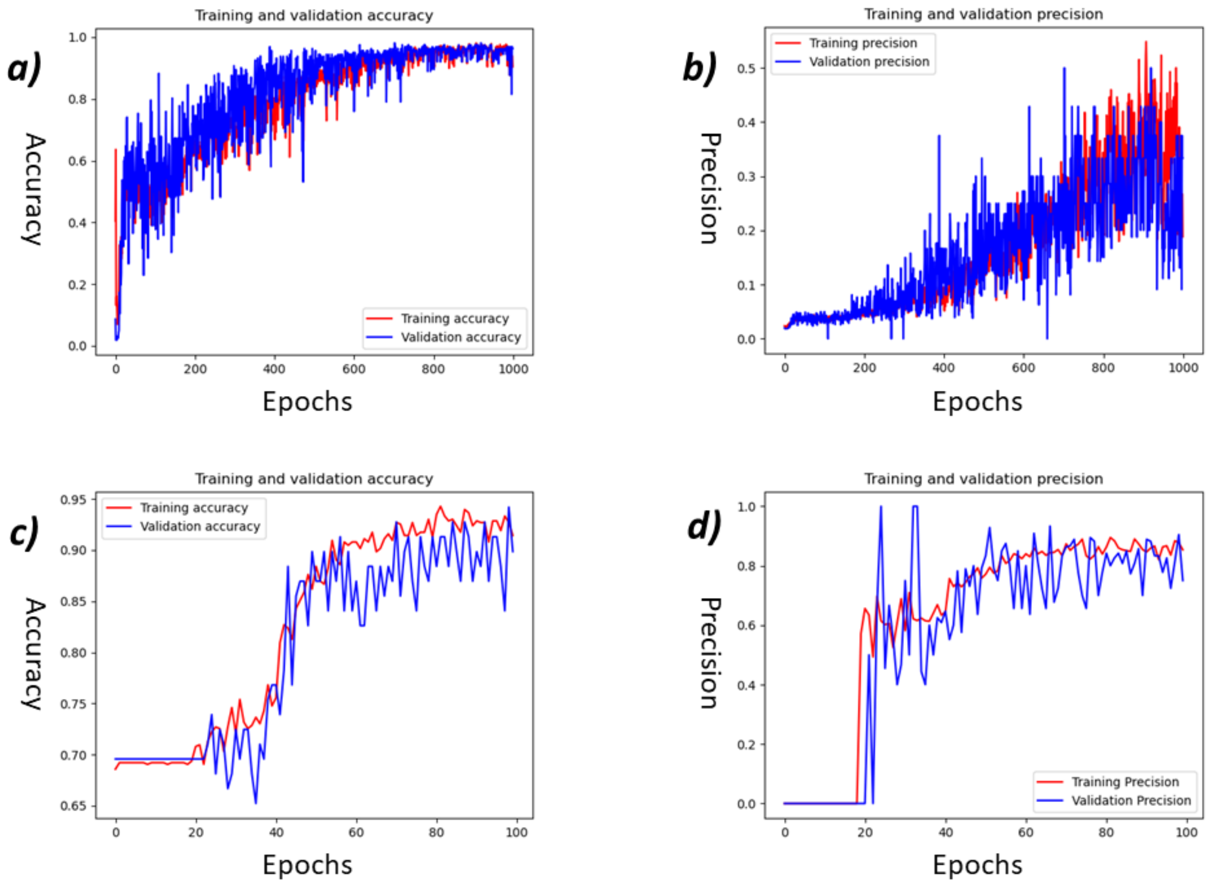

Figure S5: Training plots for the Cu(111) low CO/Cu coverage dataset over 1000 epochs with a) and b) showing accuracy and precision respectively. Plots c) and d) show similar training plots for the Si(111) - 7×7 surface for a working run over 100 epochs.

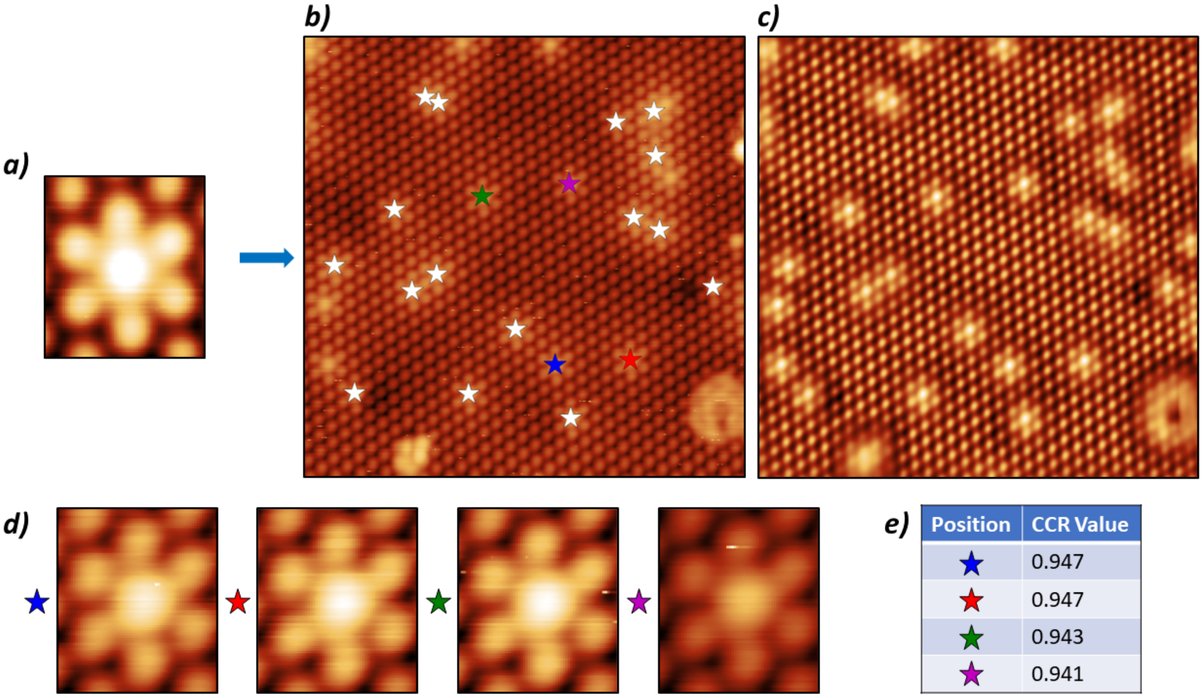

Figure S6: Cross-correlation method as applied to B:Si(111) - $(\sqrt{3} \times \sqrt{3})R30^{\circ}$. a) The reference image used comprised of a tight cropping of a dangling bond surrounded by 6 surface atoms. b) The input image, over which the reference image was scanned. c) The CCR feature map generated by scanning the reference image (a) over image b). The stars overlaid on b) show the top 20 highest correlated positions, which correspond to the peaks in c). d) The top 4 highest correlated positions with the coloured stars corresponding to the same colours in b). e) The CCR values obtained for the areas shown in d).

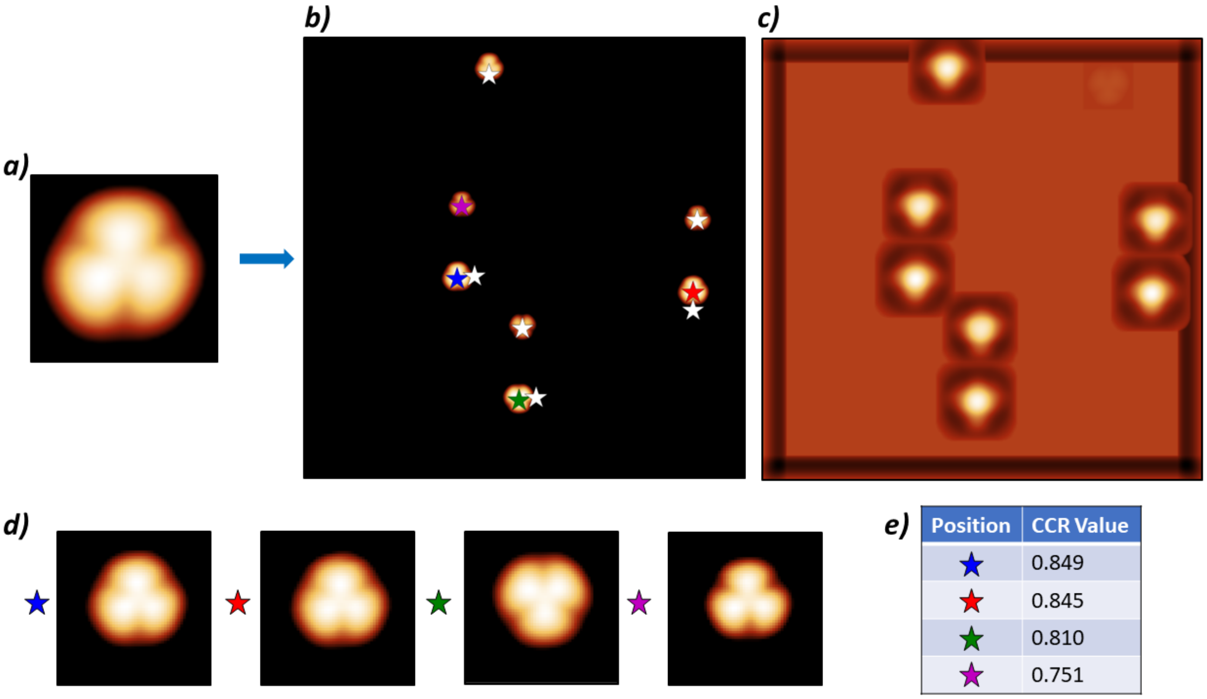

Figure S7: Cross-correlation method as applied to Cu(111) surface with a low coverage of C_{60} molecules. a) The reference image used, in this case the chosen image is a single C_{60} molecule. b) The input image, over which the reference image was scanned. c) The CCR feature map generated by scanning the reference image (a) over image b). The stars overlaid on b) show the top 10 highest correlated positions, which correspond to the peaks in c). d) shows the top 4 highest correlated positions with the coloured stars corresponding to the same colours in b). e) shows the CCR values obtained for the areas shown in d).

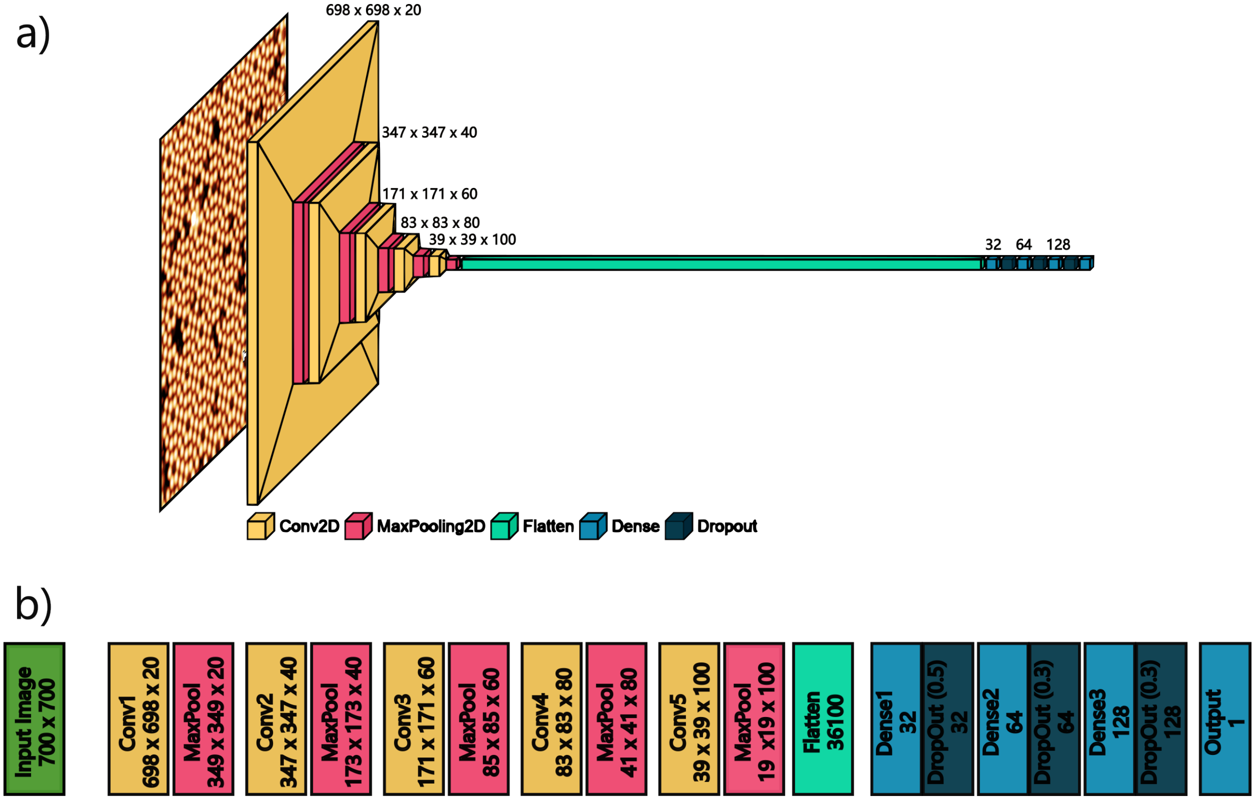

Figure S8: Architecture of the CNN model used for both the Si(111) - 7×7 and B:Si(111) datasets. It consists of a total of 18 layers: 5 consecutive convolutional-pooling layers, a flattening layer, 3 training-dropout layers, and ending with a single dense output layer.

and 259 for the B:Si(111)) using the classifier, which, for a binary classifier, will result in predictions in the form of confidences between $0 - 1$. By varying the confidence threshold at which our classifier predicts an image to belong to a specific class, and calculating the true positive and false positive rates at each, the ROC curve was produced. This curve is shown in Figure S9, where the area under the ROC curve (AUROC) corresponds to the performance. For a perfect classifier, the AUROC will be 1, whereas random guessing would be expected to give a value of 0.5 (indicated by the straight diagonal line in Figure S9).

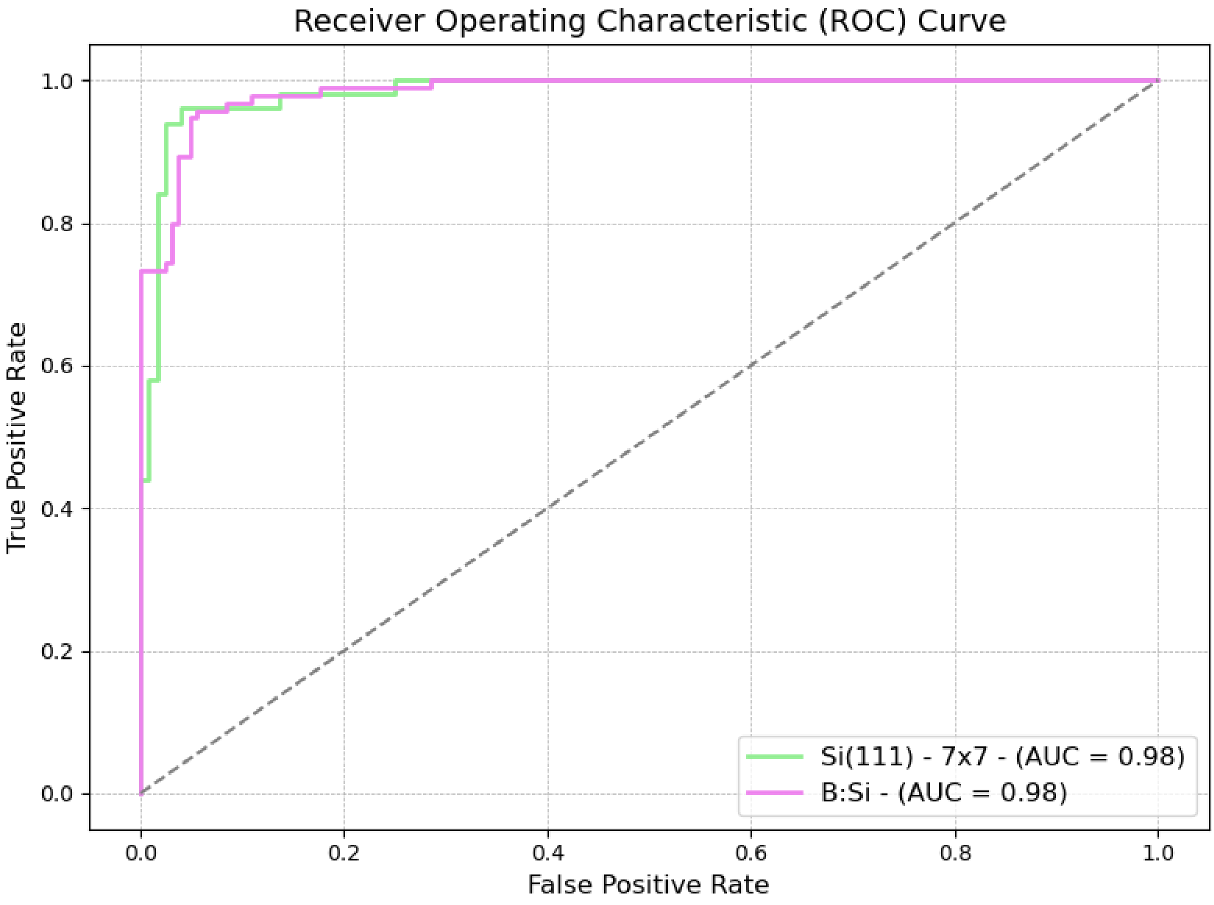

Figure S9: Receiver Operator Characteristic graphs showing the overall performance of the CNN models. The green curve shows the results for the Si(111) - 7×7 dataset, and the pink shows the B:Si(111) dataset. AUROC for both model is calculated as 0.98.

Both models yielded an AUROC of 0.98, which suggests a robust predictive performance.

Table S2: Table of results from 20 tip preparation runs used to evaluate the tool. Runs 10 and 14 are seen as outliers as the script was unable make a positive classification due to the surface. Figures given in the main text used the average excluding these runs.

Run Number	Time Taken (minutes)	Shaping Attempts
1	1.6	1
2	8.6	9
3	11.2	12
4	5.1	5
5	2.6	2
6	2.5	2
7	2.6	2
8	10.4	11
9	13.8	15
10	81.6	93
11	16.0	12
12	1.9	1
13	2.6	2
14	131.3	150
15	1.7	1
16	27.6	31
17	41.5	47
18	33.8	38
19	9.8	18
20	11.3	12
Average	19.4	23
Average Excluding 10,14	9.3	12

Tip Preparation Tool

The tip preparation tool, discussed in the main text, was tested for a total of twenty runs in order to evaluate the average time taken to fully prepare a probe tip from a "Bad" to a "Good" state. All of these runs were carried out on the Si(111) - 7×7 surface. The results from this evaluation are shown in Table S2.

The average time taken to prepare the tip over the full twenty runs was 19.4 minutes which corresponds to around 23 tip shaping attempts. Two of the runs, numbers 10 and 14, took significantly longer to achieve a "Good" tip state when compared to other runs, and so an additional average was calculated excluding these runs. This resulted in a corrected average time taken of 9.3 minutes which corresponds to roughly 12 shaping attempts. The

two outlier runs were much slower at achieving a “Good” tip due to the state of the sample itself; the distance between steps on this sample was around 50 nm, compared to the usual terrace width on the order of 100s of nm. This meant that tip changes could result in the scan area moving enough to include an area containing a step edge which would cause the image flattening procedure to change the image enough that CCR was not able to make an accurate classification.

References

(1) Yothers, M. P.; Browder, A. E.; Bumm Homer L, L. A. Real-space post-processing correction of thermal drift and piezoelectric actuator nonlinearities in scanning tunneling microscope images. *Review of Scientific Instruments* **2017**, *88*, 013708.

(2) Bert Voigtländer *Surface Characterization: A User's Sourcebook*; NanoScience and Technology; Springer Berlin Heidelberg: Berlin, Heidelberg, 2007; pp 77–81.

(3) Shi, C.; Velli, M.; Suenaga, R.; Suzuki, M.; Kakio, S.; Abe, M.; Sugimoto, Y.; Custance, O.; Morita, S. Atom tracking for reproducible force spectroscopy at room temperature with non-contact atomic force microscopy. *Nanotechnology* **2005**, *16*, 3029–3034.

(4) Saxton, W. O.; Baumeister, W. The correlation averaging of a regularly arranged bacterial cell envelope protein. *Journal of Microscopy* **1982**, *127*, 127–138.

(5) Koho, S.; Tortarolo, G.; Castello, M.; Deguchi, T.; Diaspro, A.; Vicidomini, G. Fourier ring correlation simplifies image restoration in fluorescence microscopy. *Nature Communications 2019 10:1* **2019**, *10*, 1–9.

(6) Rashidi, M.; Wolkow, R. A. Autonomous Scanning Probe Microscopy in-situ Tip Conditioning through Machine Learning. *ACS Nano* **2018**, *12*, 5185–5189.

(7) Gordon, O.; D'Hondt, P.; Knijff, L.; Freeney, S.; Junqueira, F.; Moriarty, P.; Swart, I.

Scanning Probe State Recognition With Multi-Class Neural Network Ensembles. *Review of Scientific Instruments* **2019**, *90*, 103704.

(8) Krull, A.; Hirsch, P.; Rother, C.; Schiffrin, A.; Krull, C. Artificial-intelligence-driven scanning probe microscopy. *Communications Physics* **2020**, *3*, 1–8.
